# The Golden Sheen Retina Fades by Dusk: An Incidental Adult Presentation of Oguchi Disease

**DOI:** 10.7759/cureus.112119

**Published:** 2026-07-06

**Authors:** Mohzina P Jaleel, Alfia Rasheed

**Affiliations:** 1 Ophthalmology, Travancore Medicity Medical College Hospital, Kollam, IND; 2 Ophthalmology, Amrita Institute of Medical Sciences and Research Centre, Ernakulam, IND

**Keywords:** congenital stationary night blindness, fundus discoloration, mizuo-nakamura phenomenon, oguchi disease, retinal dystrophy

## Abstract

Oguchi disease is a rare form of congenital stationary night blindness characterized by a distinctive golden-yellow or silver-gray fundus discoloration that disappears after prolonged dark adaptation, known as the Mizuo-Nakamura phenomenon. Most patients present during childhood with nyctalopia; however, asymptomatic adult presentations are uncommon. We report the case of a 39-year-old woman who underwent routine ophthalmic evaluation without any visual complaints or history of night blindness. Fundus examination revealed a diffuse golden metallic sheen involving both retinas. Following prolonged dark adaptation, the fundus reverted to a normal appearance, demonstrating the characteristic Mizuo-Nakamura phenomenon and confirming the diagnosis of Oguchi disease. This case highlights an atypical incidental adult presentation and emphasizes the importance of recognizing characteristic fundus findings even in the absence of classic symptoms.

## Introduction

Oguchi disease is a rare autosomal recessive subtype of congenital stationary night blindness characterized by a distinctive golden-yellow or silver-gray metallic discoloration of the fundus under light-adapted conditions. This abnormal appearance disappears after prolonged dark adaptation, a hallmark finding known as the Mizuo-Nakamura phenomenon. The condition is primarily associated with mutations in the SAG and GRK1 genes, resulting in impaired deactivation of photoactivated rhodopsin and delayed recovery of rod photoreceptors [1-3].

Unlike progressive inherited retinal dystrophies, Oguchi disease is a non-progressive disorder with a generally favorable visual prognosis. Accurate recognition of its characteristic clinical features is important to avoid misdiagnosis, unnecessary investigations, and inappropriate counseling regarding disease progression. Although patients typically present during childhood with nyctalopia due to rod photoreceptor dysfunction, asymptomatic cases diagnosed incidentally in adulthood are uncommon and may pose a diagnostic challenge [4,5]. Recognition of the characteristic fundus appearance and its reversibility after dark adaptation remains crucial for establishing the diagnosis, particularly in patients without classic symptoms. We report an incidental diagnosis of Oguchi disease in an asymptomatic adult female identified during a routine ophthalmic examination.

## Case presentation

A 39-year-old woman presented to the ophthalmology outpatient department of a tertiary care center for routine ocular evaluation. She was a known case of diabetes mellitus on treatment for the preceding three years. The patient denied blurring of vision, redness, photophobia, ocular discharge, or difficulty with night vision. There was no significant ocular history and no family history suggestive of night blindness.

Best-corrected visual acuity was 6/6 for distance and N6 for near vision in both eyes. Intraocular pressure measured by Goldmann applanation tonometry was 17 mmHg in the right eye and 20 mmHg in the left eye. Color vision testing was normal bilaterally. Anterior segment examination using slit-lamp biomicroscopy revealed no abnormalities.

**Figure 1:**
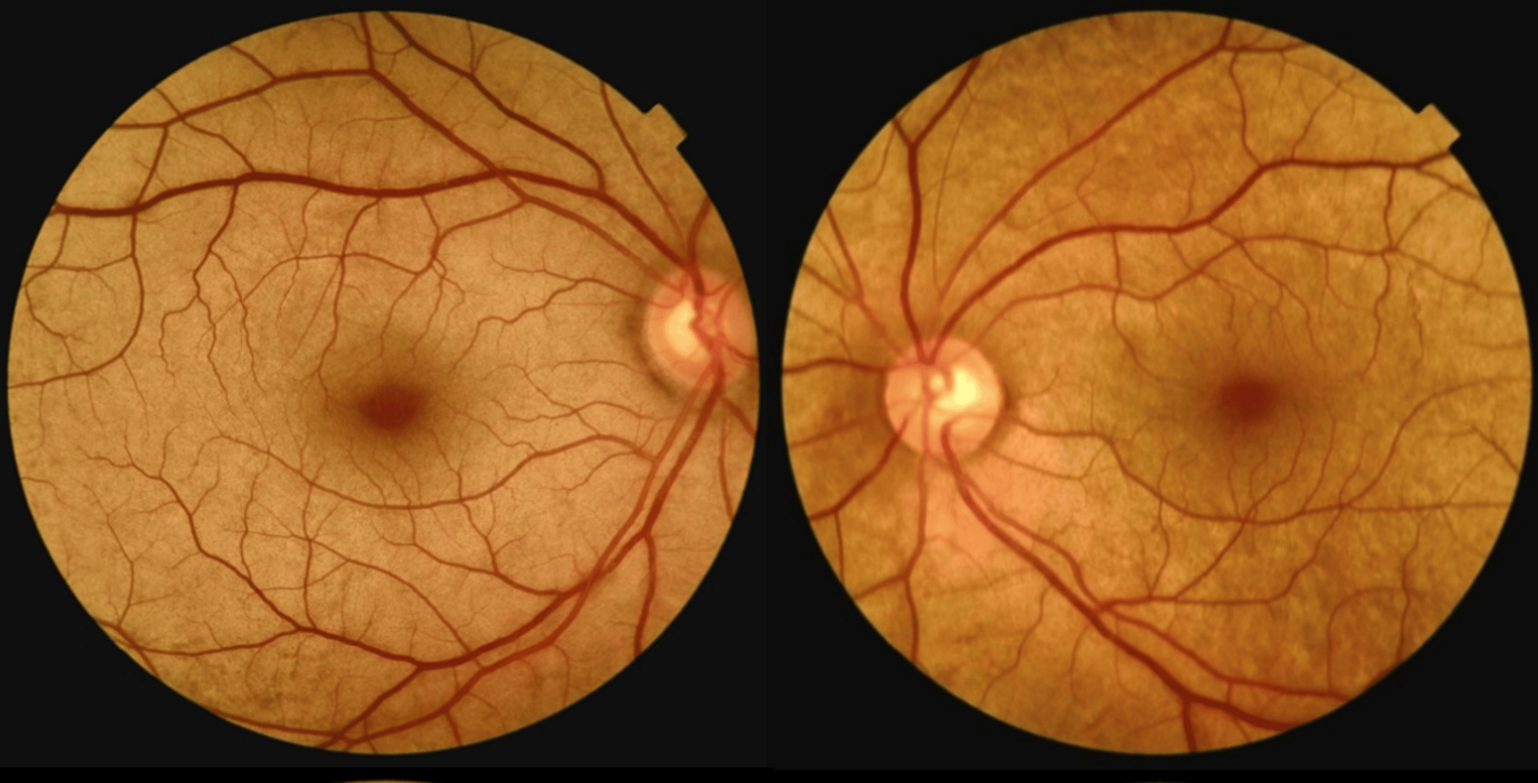
Golden fundus. Color fundus photographs showing a diffuse golden metallic sheen involving the retina of both eyes under light-adapted conditions.

Dilated fundus examination revealed a diffuse golden-yellow metallic sheen throughout the fundus, with a normal-appearing optic disc and retinal vasculature, raising clinical suspicion of Oguchi disease (Figure 1).

**Figure 2:**
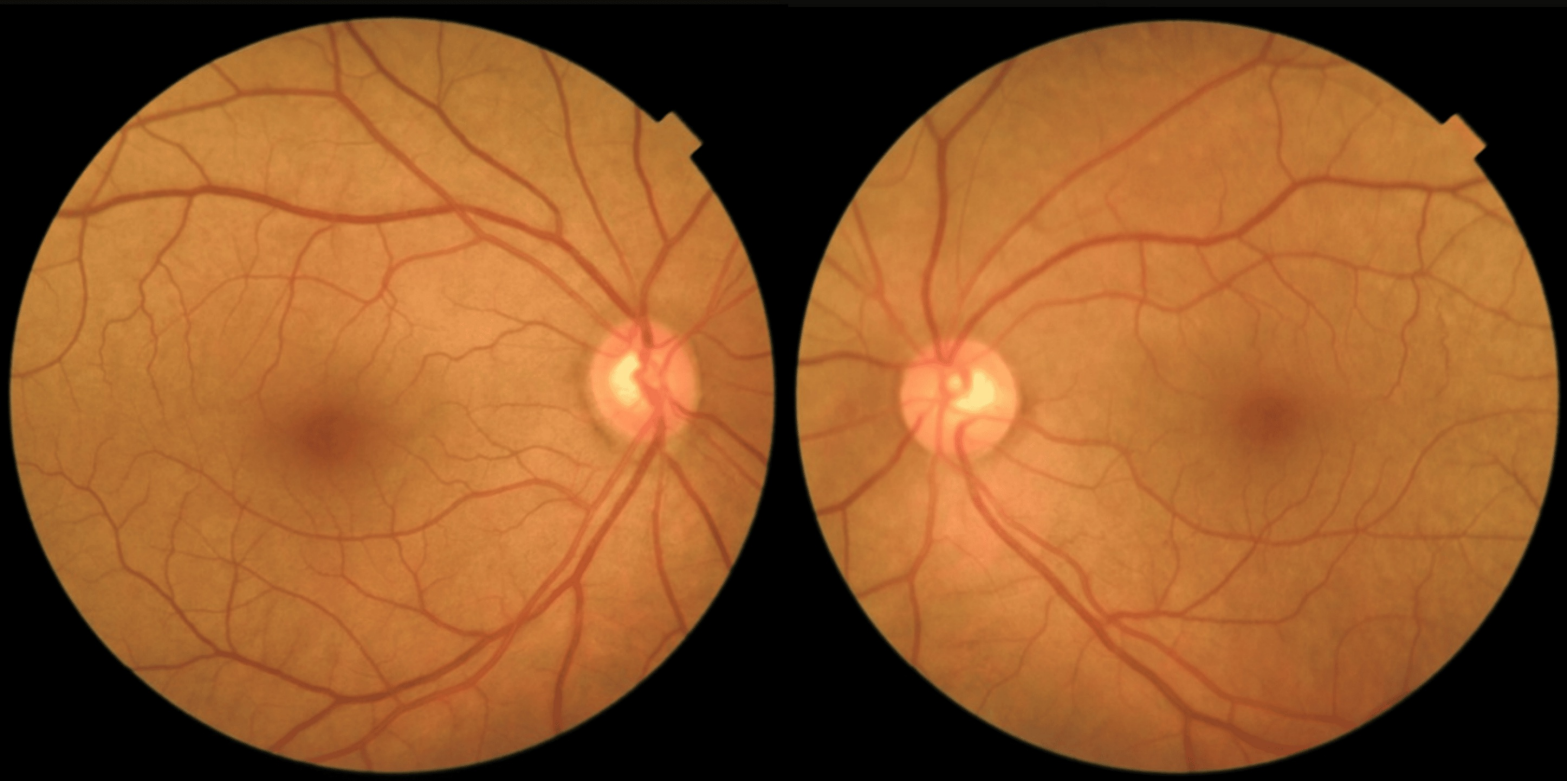
Mizuo-Nakamura phenomenon. Color fundus photographs of the right eye (left panel) and left eye (right panel) obtained after prolonged dark adaptation (approximately eight hours), demonstrating the disappearance of the metallic golden fundus sheen and the restoration of a near-normal retinal appearance, consistent with the Mizuo-Nakamura phenomenon.

To further support the clinical diagnosis, prolonged dark adaptation was performed by keeping the patient in complete darkness overnight (approximately eight hours). Following dark adaptation, the characteristic disappearance of the metallic golden fundus sheen (Mizuo-Nakamura phenomenon) was observed, strongly supporting the clinical diagnosis of Oguchi disease. Although electroretinography (ERG), genetic testing, spectral-domain optical coherence tomography (SD-OCT), and fundus autofluorescence (FAF) were not performed, the diagnosis was considered to be strongly supported by the characteristic clinical findings. Fundus examination the following morning after pharmacologic dilation showed complete disappearance of the metallic sheen, with restoration of a near-normal retinal appearance (Figure 2). This reversible change represented the classic Mizuo-Nakamura phenomenon, a pathognomonic feature of Oguchi disease [1-3].

SD-OCT, FAF, ERG, and genetic testing were not performed because the patient declined additional investigations due to financial constraints. The diagnosis was established based on the characteristic clinical features, including the Mizuo-Nakamura phenomenon, which were considered sufficient for a clinical diagnosis of Oguchi disease. Nevertheless, the absence of SD-OCT, FAF, ERG, and genetic confirmation represents a limitation of this report, as these investigations could have provided additional structural and functional corroborative evidence.

## Discussion

Oguchi disease is a rare inherited retinal disorder characterized by a distinctive golden-yellow or silver-gray fundus discoloration that reverses after prolonged dark adaptation, known as the Mizuo-Nakamura phenomenon [1]. The underlying pathophysiology involves mutations in the SAG and GRK1 genes, which correspond to Oguchi disease type 1 and type 2, respectively. These mutations impair the deactivation of photoactivated rhodopsin, leading to delayed rod photoreceptor recovery; although both subtypes share similar clinical features, they may exhibit differences in rod-recovery kinetics during dark adaptation [2,3]. Although nyctalopia is considered the hallmark symptom of Oguchi disease, asymptomatic individuals have also been reported [4]. The absence of subjective night blindness in our patient may reflect variable phenotypic expression and adaptive mechanisms within the visual system.

Although nyctalopia is considered the hallmark symptom of Oguchi disease, asymptomatic individuals have been reported in the literature [4]. The absence of subjective night blindness in our patient may reflect variable phenotypic expression and adaptive mechanisms within the visual system.

Adult diagnosis without significant visual complaints is uncommon but has been previously described [5]. Recognition of characteristic fundus findings is therefore essential, particularly when patients present for routine ophthalmic evaluation.

The differential diagnosis of Oguchi disease includes conditions that produce a tapetal or metallic fundus reflex, such as X-linked retinoschisis, ocular siderosis, and other tapetal-like retinal reflexes. However, the characteristic disappearance of the metallic sheen after prolonged dark adaptation (the Mizuo-Nakamura phenomenon) reliably distinguishes Oguchi disease from these conditions [1].

Recent case reports have also highlighted atypical presentations of Oguchi disease and emphasized the importance of recognizing the condition even in the absence of classical symptoms or with unusual genetic variants [6].

The coexistence of diabetes mellitus in the present case appears incidental, as no established association between diabetes mellitus and Oguchi disease has been reported.

## Conclusions

Oguchi disease should be considered in patients demonstrating a characteristic golden metallic fundus, even in the absence of nyctalopia or visual complaints. Recognition of the Mizuo-Nakamura phenomenon remains critical for diagnosis and can prevent unnecessary investigations. This case highlights a rare incidental adult presentation of Oguchi disease and underscores the importance of careful fundus examination during routine ophthalmic evaluation.
